# T cell biology in neuromuscular disorders: a focus on Duchenne Muscular Dystrophy and Amyotrophic Lateral Sclerosis

**DOI:** 10.3389/fimmu.2023.1202834

**Published:** 2023-10-18

**Authors:** Julia Pereira Lemos, Liliane Patrícia Gonçalves Tenório, Vincent Mouly, Gillian Butler-Browne, Daniella Arêas Mendes-da-Cruz, Wilson Savino, Piera Smeriglio

**Affiliations:** ^1^ Sorbonne Université, INSERM, Institut de Myologie, Centre de Recherche en Myologie, Paris, France; ^2^ Laboratory of Cell Biology, Institute of Biological and Health Sciences, Federal University of Alagoas, Maceio, Alagoas, Brazil; ^3^ Laboratory on Thymus Research, Oswaldo Cruz Institute, Oswaldo Cruz Foundation, Rio de Janeiro, Brazil; ^4^ National Institute of Science and Technology on Neuroimmunomodulation (INCT-NIM), Oswaldo Cruz Institute, Oswaldo Cruz Foundation, Rio de Janeiro, Brazil; ^5^ Rio de Janeiro Research Network on Neuroinflammation (RENEURIN), Oswaldo Cruz Institute, Oswaldo Cruz Foundation, Rio de Janeiro, Brazil; ^6^ INOVA-IOC Network on Neuroimmunomodulation, Oswaldo Cruz Institute, Oswaldo Cruz Foundation, Rio de Janeiro, Brazil

**Keywords:** Duchenne Muscular Dystrophy, Amyotrophic Lateral Sclerosis, effector T cells, regulatory T cells, immunotherapy

## Abstract

Growing evidence demonstrates a continuous interaction between the immune system, the nerve and the muscle in neuromuscular disorders of different pathogenetic origins, such as Duchenne Muscular Dystrophy (DMD) and Amyotrophic Lateral Sclerosis (ALS), the focus of this review. Herein we highlight the complexity of the cellular and molecular interactions involving the immune system in neuromuscular disorders, as exemplified by DMD and ALS. We describe the distinct types of cell-mediated interactions, such as cytokine/chemokine production as well as cell-matrix and cell-cell interactions between T lymphocytes and other immune cells, which target cells of the muscular or nervous tissues. Most of these interactions occur independently of exogenous pathogens, through ligand-receptor binding and subsequent signal transduction cascades, at distinct levels of specificity. Although this issue reveals the complexity of the system, it can also be envisioned as a window of opportunity to design therapeutic strategies (including synthetic moieties, cell and gene therapy, as well as immunotherapy) by acting upon one or more targets. In this respect, we discuss ongoing clinical trials using VLA-4 inhibition in DMD, and in ALS, with a focus on regulatory T cells, both revealing promising results.

## 1 Introduction

### 1.1 Neuromuscular diseases: a focus on DMD and ALS

Neuromuscular disorders (NMD) encompass a vast number of pathological conditions affecting the nervous system and the skeletal muscle and their communication, frequently causing muscle wasting and weakness. Some NMD, such as Duchenne Muscular Dystrophy (DMD), have a defined genetic origin and others, like Amyotrophic Lateral Sclerosis (ALS), can have a genetic origin or arise from sporadic mutations.

DMD is a genetic disorder due to mutations in the gene encoding the dystrophin protein. It is an X-linked disease mainly affecting boys, with a relatively high incidence (1/1500 boys born alive) It is characterized by continuous cycles of skeletal muscle degeneration and regeneration ultimately resulting in muscle wasting and loss of ambulation between 8-14 years of age (1). It has been shown that intramuscular inflammatory infiltrates, which are found in DMD patients, are mainly composed by T lymphocytes (especially CD8+ T cells) and macrophages (2). The most commonly used DMD model is the C57BL/10ScSn (mdx) mouse, which carries a naturally occurring point mutation in exon 23 of the dystrophin gene, an absence of the full-length Dp427 protein isoform (3, 4) and to a lesser extent genetic variation of the mdx model (5–7). Other DMD murine models include the D2.mdx mouse, corresponding to the mdx mice bred on the DBA2/J background (8–10), and the dystrophin/utrophin double-knockout (dko) mouse (11–14), among others.

ALS is a neurological disorder characterized by the loss of motor neurons (MN) and paralysis of the skeletal musculature (15). It affects mostly middle-aged individuals (16), being the most common MN disorder in adults (17). The disease is characterized by muscular weakness due to progressive atrophy leading to paralysis and death, 3 to 5 years after diagnosis (18), secondary to respiratory failure (19). The diagnosis of ALS is clinical, based on the patient’s history and symptomatic examination, usually supported by electrophysiological investigation (20). Around 10% of the ALS cases have a familial history (fALS), caused by mutations in one or several genes, mostly inherited in an autosomal dominant pattern (21). Of these, around 20% are associated with mutations in the Cu/Zn superoxide dismutase 1 (mSOD1) gene and approximately 40% in the chromosome 9 open reading frame 72 (C9orf72) gene, in Europe and the United States, and can drastically vary, notably in Asian patients (21–23). Experimental models for both mutations have been developed, namely the mSOD1 and the C9orf72 mice (24) that despite some limitations, recapitulate some of the main pathophysiological aspects of the disease.

### 1.2 General features of T-cell development and generation of regulatory T cells

T cells develop in the thymus, from common lymphoid progenitors of bone marrow origin. These precursors migrate into the bloodstream and enter the thymus through vessels present at the corticomedullary junction of the thymic lobules, where they receive a signal from the microenvironment that activates specific genes, leading to commitment to the T cell lineage (25).

T cells maturation occur via positive and negative selection processes occurring through the interaction of the T-cell receptor (TCR) with class I or class II major histocompatibility complex (MHC) molecules associated with self-peptides. Those complexes are expressed by the thymic epithelium (TEC) and thymic dendritic cells (DC) (25) and largely prevent the development of cells with autoimmune potential. During the double-negative (DN; CD4^-^CD8^-^) stage, cells undergo gene rearrangement of the β chain or the γδ chains of the T-cell receptor, determining whether the thymocytes will be αβ or γδ, although most develop into αβ (25). Cells that do not successfully rearrange die; in contrast, cells that undergo a successful rearrangement proliferate intensively. At this stage, cells express a pre-TCR that is associated with the CD3 molecular complex necessary for receptor signaling. Pre-TCR expression leads to cell proliferation, arrest of rearrangement of β-chain genes and expression of CD4 and CD8 coreceptors, determining the double-positive stage (DP; CD4^+^CD8^+^), which represents about 80% of thymocytes (25). At this stage, the rearrangement of the α chain genes begins, leading to the expression of the definitive TCRαβ complex, making DP cells responsive to specific antigens and then subject to positive or negative selection processes (25). Cells whose TCRs are able to bind pMHC with moderate avidity are positively selected, following the differentiation process (26). Thymocytes that express TCRs associated with MHC class I-bound self-peptides loose expression of the CD4 molecule and become TCR^hi^CD4^-^CD8^+^ (about 5%), whereas those that express TCRs associated with MHC class II become TCR^hi^CD4^+^CD8^-^ (about 10%). At this final stage of intrathymic T-cell development, the single-positive (SP) thymocytes are now mature cells ready to leave the thymus and exert their effector and regulatory functions in the periphery of the immune system. In fact, some clones that recognize self-antigens with high avidity differentiate into CD4^+^CD8^-^CD25^+^Foxp3^+^ regulatory T cells (Treg), through a mechanism that appears to be dependent on the avidity and duration of TCR signaling and TGF-β and cytokine-mediated survival, such as IL-2, IL-7, and IL-15 (27, 28). Treg cells are CD4^+^ T lymphocytes, characterized by the expression of the transcription factor FOXP3, surface expression of CTLA-4, the glucocorticoid-induced TNF receptor family related protein (GITR) and CD25 (the IL-2 receptor alpha chain).

Tregs can also differentiate from CD4^+^ T cells in the periphery of the immune system (peripheral Tregs) or within the thymus (central Tregs); the latter maturing under the direct influence of the thymic microenvironment (29). These thymus-derived cells represent the majority of Tregs in the periphery compared to Tregs differentiated from conventional naïve T cells (30).

In the periphery, after encountering the specific antigen and being activated, naïve T CD4^+^ cells even differentiate into types 1, 2 or 17 T-helper (Th) cells, among others, depending on the stimulus they receive. Although morphologically indistinguishable, these cells present distinct patterns of secreted cytokines and, consequently, specific responses (31).

CD8^+^ T cells, also known as cytotoxic T lymphocytes, are very important in the immune defense against intracellular pathogenic agents and also in the surveillance of tumor formation, acting through the induction of the cell death pathway (apoptosis) in the target cell (32).

## 2 T-cell immune responses in Duchenne Muscular Dystrophy

**Figure 1:**
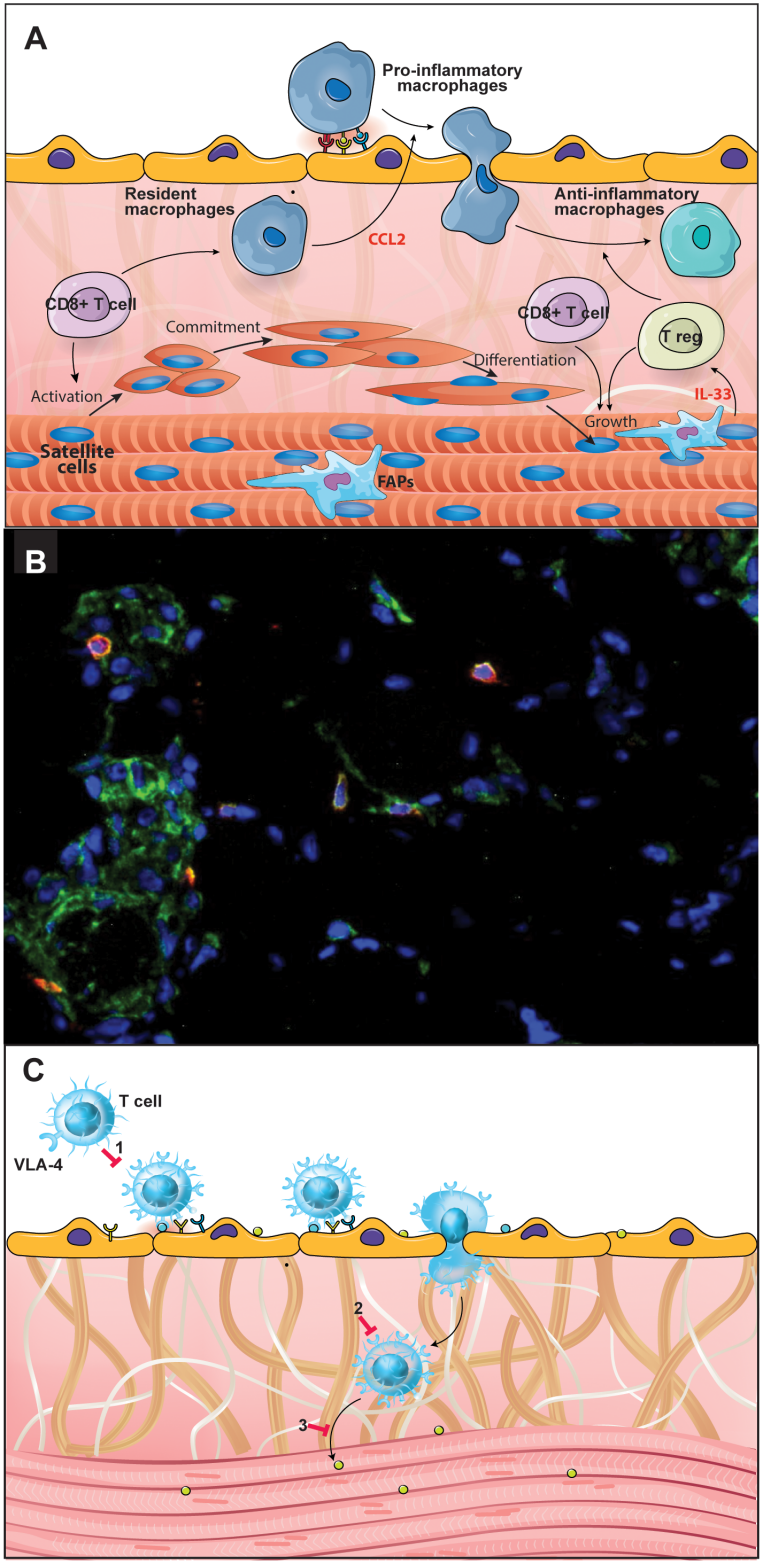
CD49d^high^ T lymphocytes are involved in the Duchenne Muscular Dystrophy (DMD) pathophysiology. Effector T cells are important in DMD pathophysiology and progression, with a special role for the CD49d^high^ T cell subpopulations. **(A)** During the post-injury phase of muscle repair, macrophages initially acquire a pro-inflammatory phenotype followed by a switch into anti-inflammatory macrophages, necessary to solve inflammation and promote myofiber growth. These events are crucial for the regenerative process and depend on T cells, notably CD8^+^ and regulatory T cells. **(B)** Immunostaining in a quadriceps muscle biopsy obtained from a DMD patient at the onset of the disease, showing the *in situ* detection of CD8^+^ (green) CD49d^+^ (red) cells. Nuclei were visualized using DAPI (blue). **(C)** The scheme illustrates the notion that CD49d inhibitors can be envisioned as a therapeutic approach to decrease inflammation-mediated tissue damage, by blocking VLA-4-mediated T lymphocyte transendothelial migration; (1) as well as the intramuscular migration of the cells through a fibronectin-containing extracellular network; (2) and the CD49d^high^ T cell adhesion to myoblasts/myotubes, (3) with consequent inhibition of muscle cell apoptosis. Figure created using the Adobe Illustrator software. Immunofluorescence micrography gently provided by Dr. Fernanda Pinto Mariz (Pediatric Institute, Federal University of Rio de Janeiro, Brazil).

Several studies have aimed at clarifying the participation of the immune system in the pathophysiology of DMD. During the disease process, immediately after muscle damage, degenerating muscle fibers release Danger Associated Molecular Patterns (DAMP), in addition to pro-inflammatory cytokines and chemokines which attract innate immune cells. Thus, the muscle is first invaded by mast cells and neutrophils and a pro-inflammatory response dominates the scene with the stimulation of pro-inflammatory macrophages and proliferation of Th1 lymphocytes (33). During muscle repair, macrophages first acquire a pro-inflammatory phenotype. In this phase, proliferation of myoblast progenitors and initial differentiation occurs, followed by a switch from proinflammatory into anti-inflammatory macrophages, necessary to resolve inflammation and promote myofiber growth, as shown by *in vivo* and *ex vivo* analysis using a murine model (33, 34). These events are crucial for the whole regenerative process and depend on T cells. In fact, it has been shown that genetic deletion of CD8^+^ T lymphocytes impairs pro-inflammatory macrophage infiltration due to the absence of the chemokine CCL2 and is likely to be related to a defective muscle regeneration in a cardiotoxin-induced mouse skeletal muscle injury/regeneration model (35). These data indicate the importance of chemokines secreted by CD8^+^ T cells upon monocyte/macrophage migration to the muscle (**Figure 1A**). Moreover, Rag2^–^Il2rb^–^Dmd^–^ immunodeficient mice, which lack T, B, and NK cell activity, presented a delayed degeneration/regeneration process when compared with the immunocompetent mdx mice (36).

In DMD it has been proposed that the lack of dystrophin has a consequence on muscle cell injury resulting in the release of intramuscular antigens that can be specifically recognized by cells of the adaptive immune response, such as B and T lymphocytes (37). Accordingly, T cells detected within the DMD muscle tissue predominantly express TCR belonging to the Vβ2 family (38). In particular, Vβ expansions point to T-cell clones that are selected during these immune responses, suggesting that T-lymphocytes expressing Vβ2 are involved in an antigen-driven late effector cell-mediated immune response. Moreover, most DMD patients exhibit a conserved sequence of four amino acids in the complementarity-determining region (CDR) 3 of the Vβ2 TCR, further suggesting specific recognition of muscle antigens (38, 39). Similarly, in the mdx mouse, T cells expressing Vβ8.1/8.2 predominated the pool of all TCR-Vβ^+^ expressing cells. Moreover, the Vβ8.1/8.2-expressing T cells were not present in the spleen, suggesting they may expand in the dystrophic mdx muscle (40). It should be noted that DMD muscle fibers were invaded by CD8^+^ T cells expressed MHC class I molecules on their surfaces (41). Expression of MHC I is increased in dystrophic muscle as observed in myofibers invaded by CD8^+^ T cells compared to muscle from healthy individuals, which have little expression of MHC I (41, 42). Once activated, cytotoxic CD8^+^ T lymphocytes could then migrate and recognize specific peptides on the surface of muscle fibers triggering the release of death-related molecules including perforin, granzyme and tumor necrosis factor (TNF)-α, resulting in consequent tissue injury (43).

The analysis of a cohort of 75 DMD patients, has revealed that there is an increase in the percentage of circulating CD4^+^ and CD8^+^ T lymphocytes expressing high levels of CD49d (the alpha chain of the VLA-4 integrin) and this is positively correlated with a faster disease progression. Furthermore, T cells from patients with a more rapid disease progression exhibited higher trans-endothelial and fibronectin-driven migratory responses when compared to healthy individuals (44). The DMD patients with poorest prognosis who lost ambulation before ten years of age included in this study showed an increase in VLA-4 expression on T cells and a higher expression of the interstitial VLA-4 ligand, fibronectin, within the muscle. Furthermore, CD49d^+^CD4^+^CD3^+^ as well as CD49d^+^CD8^+^CD3^+^ T lymphocytes were seen intermingled with the fibronectin-containing network of the injured muscle (seen in **Figure 1B**). Lastly, T cells from DMD patients adhered more strongly to myotubes compared to circulating T cells from healthy subjects (44). Similar results were found in an animal model for DMD, namely the Golden Retriever Muscular Dystrophy dog. Higher numbers of circulating CD4^+^CD49d^high^ T cells at early stages of the disease were found to be highly predictive for the loss of ambulation of the dogs before 6 months of age (45).

Increased expression of interferon (IFN)-γ, transforming growth factor (TGF)-β and chemokines, such as CCL14, CCL2, CXCL12 and CXCL14 has also been observed in the muscle of DMD patients (46). Considering that extracellular matrix (ECM) elements can interact directly with immune cells functioning as a substrate for binding soluble factors such as cytokines and chemokines, it is plausible to think that a combined action involving an increase in fibronectin expression and in chemokines/cytokines could enhance the recruitment of inflammatory cells to this area, thus maintaining and eventually enhancing inflammation and fibrosis.

As mentioned above and taking into account the studies performed in DMD patients, it is conceivable that the muscle damage initially caused by the absence of dystrophin could result in the exposure of antigens on the surface of muscle fibers. Activated T lymphocytes with high expression of CD49d (CD49d^high^) could migrate to muscle tissue directed by a chemotactic/haptotactic gradient. Moreover, CD49d^high^ T cell subsets obtained from DMD patients exhibited a higher *in vitro* migration capacity across endothelia and through fibronectin, when compared to CD49d^low^ T cell subpopulations from healthy donors (44). Once within the muscular tissue, CD8^+^CD49d^high^ T lymphocytes could recognize antigens on the surface of the fibers causing their destruction.

Overall, effector T cells are important in DMD pathophysiology (43) and disease progression with a special role for the CD49d^high^ T cell subpopulations, which in turn can be used as a prognostic marker of disease progression (44). Moreover, CD49d inhibitors can be envisioned as a therapeutic approach to decrease inflammation-mediated tissue damage (**Figure 1C**), with consequent amelioration in the quality of life of DMD patients. To test this approach a phase II clinical trial using small interfering RNA (siRNA) to inhibit CD49d expression in T cells in adolescent males aged 10 to 18 years with a diagnosis of non-ambulatory DMD for at least 3 months is ongoing (http://www.anzctr.org.au/Trial/Registration/TrialReview.aspx?ACTRN=12618000970246) (47, 48).

Within the context of T-cell biology in DMD, is also of note the presence and action of DCs, although the studies on this subject remain rather scarce. DCs are at the interface between innate and adaptive immunity, being professional antigen-presenting cells (APCs), presenting foreign antigens as peptides for T cell recognition and mediating their activation and differentiation. DCs are present in normal skeletal muscle and MHC II^+^ cells increase after injury (49). The participation of these cells to the disease process is plausible, since they express Toll-like receptor (TLR) 7, which can bind to RNA and trigger cytokine production, enhancing or at least maintaining the cycle of inflammation/degeneration/regeneration (50). Among the cytokines released, TGF-β seems to be strongly induced in symptomatic DMD patients (51, 52). It is interesting to note that, at least *in vitro*, DCs interact with myoblasts, increasing their proliferation and migration, as well as cytokine secretion by these cells (53).

## 3 Amyotrophic Lateral Sclerosis and T cell response

In contrast to DMD, ALS is a composite disease with non-cell autonomous mechanisms contributing to neurodegeneration. For instance, activation of glial cells such as microglia and astrocytes is a typical hallmark, contributing to the disease phenotype through a local inflammatory response (54). In the healthy adult brain, resting microglia monitor the surrounding microenvironment, interact with nearby cells, and play a key role in the central nervous system (CNS) development, homeostasis and repair (55–57). Through pattern recognition receptors (PRR), microglial cells sense molecules frequently found in pathogens (the Pathogen-Associated Molecular Patterns; PAMPs) or molecules released by damaged cells, the DAMPs, leading to the production of soluble molecules and modifications in their interactions with neurons (58). The accumulation of misfolded proteins in the MNs is probably one of the first events in neurodegeneration, leading to inflammation following the release of DAMPs, such as reactive oxygen species (ROS), which, in turn, activate glial and immune cells (59).

**Figure 2:**
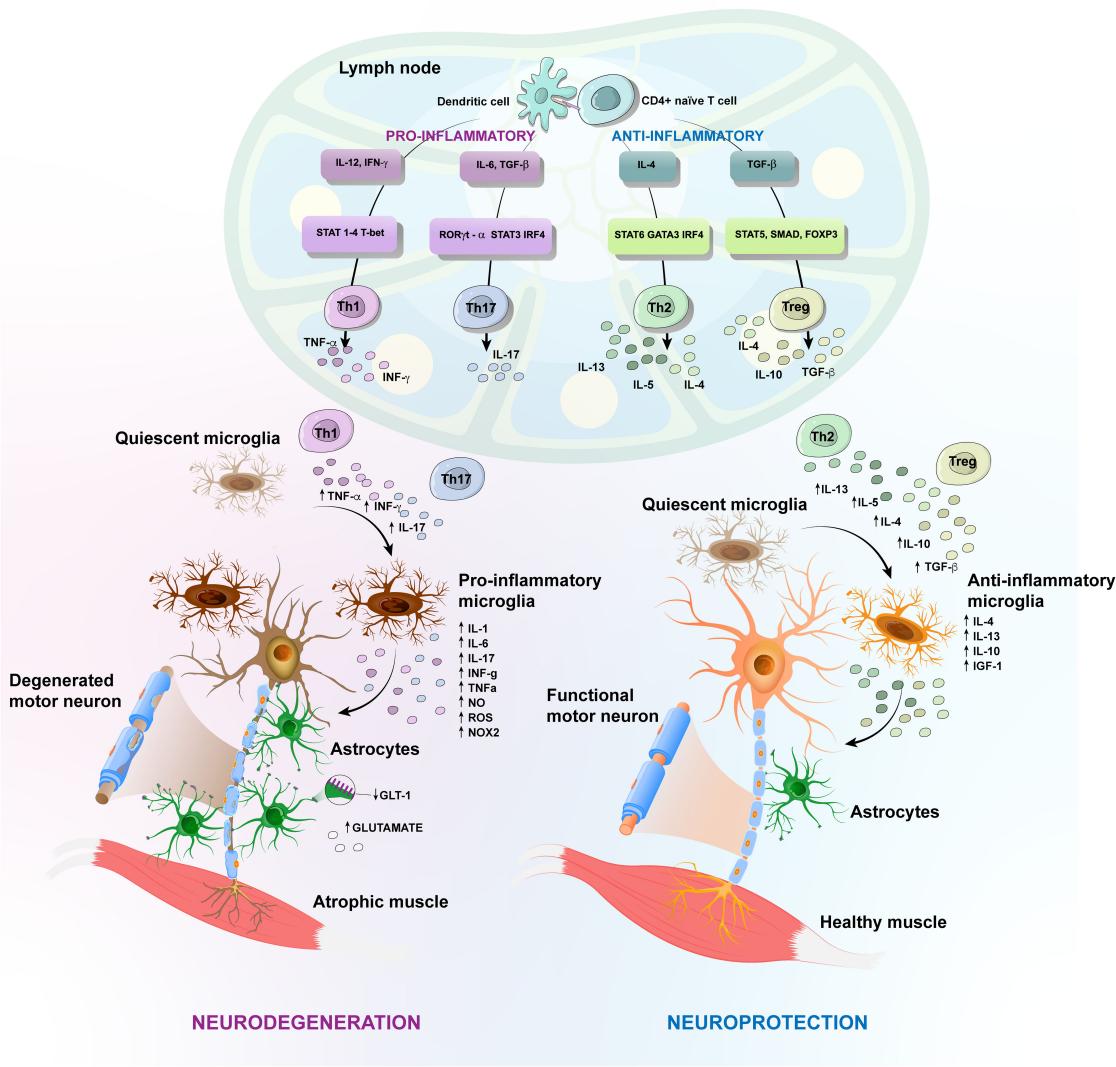
Amyotrophic Lateral Sclerosis (ALS) different protective versus harmful phenotypic responses. Schematic representation of T-cell implication in the inflammatory events in ALS caused by toxic accumulation of mutated proteins and consequent release of damage-associated molecular patterns (DAMPs). Antigens released during the axonal degeneration are carried by antigen-presenting cells, like dendritic cells, until draining lymph nodes, where they are presented to naïve T cells. The activation of CD4^+^ T cells generates different subtypes of T helper (Th) cells. In the CNS, quiescent microglia and astrocytes are activated and secrete pro-inflammatory factors, including oxygen-reactive species (ROS), tumor necrosis factor-α (TNF-α), interferon gamma (IFN-γ). These factors attract infiltrating Th1 and Th2 CD4^+^ cells, increasing inflammation by releasing TNF-α, IL-6 and IFN-γ, for instance. Likewise, the pro-inflammatory cytokines inhibit the development of the anti-inflammatory regulatory T (Treg) cells. Further, astrocytes in ALS display reduced expression of the glutamate transporter-1 (GLT-1) and fail to effectively regulate the extracellular levels of glutamate. The antigens released during axonal dismantling also activate protective responses that contribute to neuroprotection, by suppressing inflammation, cleaning cellular debris and promoting extracellular matrix rebuilding. There is activation of protective microglia, which produce anti-inflammatory cytokines, as well as neurotrophic factors and Th2 CD4^+^ cells, which in turn confer neuroprotection by producing anti-inflammatory cytokines, ultimately enhancing the neuroprotective effects of activated microglia. Anti-inflammatory Treg cells are also increased in number, favoring the neuroprotective response, by inhibiting the harmful inflammatory environment, through the secretion of IL10, IL-4 and TGF-β. Figure created using the Adobe Illustrator software.

When activated, microglia exist in a variety of phenotypic states, with two extremes, as depicted in **Figure 2**. One corresponds to a protective phenotype, which produces anti-inflammatory cytokines, such as interleukin (IL)-4, IL-13, IL-10, as well as progranulin, and neurotrophic factors, such as insulin-like growth factor (IGF)-1, which contributes to neuroprotection by suppressing inflammation, cleaning cell debris and promoting ECM reconstruction (60–62). The other extreme is a harmful phenotype, capable of producing ROS and pro-inflammatory mediators, including the subunit of nicotinamide-adenine-dinucleotide-phosphate (NADPH) oxidase (NOX) 2, IL-1α, IL-1β, IL-6, IL-12, IL-23, TNF-α, inducible nitric oxide synthase (iNOS), matrix metalloproteinase (MMP) 12 and IFNs, leading to neurodegeneration (61, 63). Studies using animal models of ALS have shown that microglial activation occurs before disease onset, concomitant with loss of the neuromuscular junctions (NMJ), and increases during disease progression (64–66). The NMJ, the functional unit of skeletal muscles, is composed of terminal axons of presynaptic MN, post synaptic skeletal muscle and associated glial cell types, the Schwann cells (67). Results from different ALS models have shown that MN degeneration begins in terminal axons and ascends to the cell body in a phenomenon called “axonal dieback” (68–70). Expression of the mutated protein only in skeletal muscles leads to abnormalities in the NMJ due to mitochondrial dysfunction and activation of cell death pathways (71). Transgenic mice engineered to overexpress mSOD1 in specific tissues show MN degeneration only when the mutant protein is expressed in both neurons and microglia or astrocytes (70, 72). Similarly, selective reduction of mSOD1 in microglia or astrocytes of ALS mice increases their survival (73, 74).

Astrocytes are the most abundant glial cells and a key component in the CNS environment, being involved in a variety of homeostatic functions, through the secretion of neurotrophic factors (75). They are capable of amplifying either neuroprotective or neurotoxic effects mediated by the microglia and can directly or indirectly influence the fate of MNs. Experiments using a cell transplantation approach have revealed a detrimental role for mSOD1-ALS astrocytes (76). In fact, reactive astrocytes surround both upper and lower degenerating MNs and several molecules have been linked to the astrocyte-mediated toxicity in ALS. For example, ALS astrocytes display a reduced ability to provide metabolic support to MNs and to effectively regulate the extracellular levels of neurotransmitters, such as glutamate. Excessive stimulation of glutamate receptors causes excitotoxicity, thereby increasing the influx of calcium, which can be toxic to neurons (**Figure 2**). Other molecules secreted by activated astrocytes that can cause neurotoxicity are pro-inflammatory cytokines and inflammatory mediators, such as NOX2, iNOS, ROS, IFN-γ, prostaglandin D2 (PGD2) and TGF-β (77–81). In fact, it has been demonstrated that astrocytes from ALS patients secrete more IL-6 and have elevated levels of NOX2, iNOS and ROS production, exacerbating the inflammatory response and MN degeneration (82, 83).

The link between microglia, astrocytes and the adaptive immune system is demonstrated by the crosstalk between these activated resident cells and the CNS-infiltrating peripheral immune components such as T cells. Accordingly, reactive microglia coupled with infiltrating T cells were found in autopsies of ALS patients (84). The CD4^+^ T cell infiltration is likely related to an increase in the chemokine CCL2 and microglial activation (85–87). Moreover, the expression of the chemokine receptors CXCR3 and CXCR4, as well as the chemokines CCL2, and CCL5, are also increased in T cells from ALS patients, compared to healthy controls (88). Recent data highlighted the protective role of these infiltrating CD4^+^ cells in ALS. During the dismantling of the neuromuscular junction, with axonal retraction and demyelination, several antigens are presented at the site of the injury, which leads to an immune response (16). However, whether the antigenic origin is neuronal or muscular is not yet known, although it seems that the debris formed during axonal disconnection from its target musculature are the source of antigens that lead to activation of a neuroprotective immune response. Consequently, there is an induction of debris clearance and an initial repair phase culminating in exacerbation of disease progression (19).

T cells isolated from the cerebrospinal fluid of ALS patients appear to have undergone clonal expansion, also suggesting antigen-mediated activation (89). Jones et al. (2015) established that MHC class II-mediated antigen presentation is necessary for CD4^+^ T cells to confer neuroprotection. Following axonal degeneration, MHC class II-expressing antigen presenting cells in the draining lymph nodes initially activate peripheral CD4^+^ T cells, which are subsequently reactivated by CNS-residing MHC class II^+^ APCs expressing the same antigen, also supporting the idea that glial cells can present antigens to T cells (90, 91).

Th2 CD4^+^ cells confer neuroprotection by producing anti-inflammatory cytokines, such as IL-4, IL-5 and IL-13, which in turn enhance the neuroprotective effects of activated microglia (92).

However, the antigen presentation following axonal disconnection leads to the differentiation of several CD4+ T cell subsets in the draining lymph nodes and a predominant Th1 and Th17 response is observed in mSOD1 mice (93). Th1 and Th17 cells are CD4^+^ T cell subtypes that promote neuroinflammation, by producing pro-inflammatory cytokines. Th1 cells produce IFN-γ that promotes activation of a harmful microglia, increasing the release of ROS and NO. In turn, the microglia exacerbate the proliferation and function of Th1 cells (66, 92, 94). Neurotoxic effects are also attributed to IL-6 released by Th1 cells. In addition, Th1 and Th17 cells produce high amounts of TNF-α. Both, IL-6 and TNF-α, are known to inhibit the development of the anti-inflammatory IL-10-producing Treg cells (95, 96).

Whether there is a difference in the number of T cells in ALS is still controversial. There are studies showing both decreased or elevated numbers of CD4^+^ T cells during disease progression in the peripheral blood of ALS patients in comparison to healthy controls (97, 98). To elucidate this question, the different CD4^+^ T cell subpopulations should be considered, since changes in the total number may correlate with modulation in specific CD4^+^ T-cell subsets rather than CD4^+^ T cells as a whole. Indeed, it seems there is a shift from a supportive Th2 to a deleterious Th1 response as the disease progresses. In this sense, some studies showed reduced numbers of CD4^+^ T cells (99) and increased proportions of proinflammatory effector T cells (100, 101) in ALS patients.

Although over the course of ALS, part of the CD4^+^ T cell response is harmful, it has been shown that total CD4^+^ T cell deletion accelerates the disease. When mSOD1 mice were crossed with T cell receptor β chain (TCRβ) deficient mice (TCRβ^-/-^; that lack TCRαβ^+^CD4^+^ and TCRαβ^+^CD8^+^ T cells), there was an increased rate of disease progression, although the time of disease onset was not modified (90). Similar results were observed when mSOD1 mice were crossed with Rag2^−/−^ mice (lacking B and T lymphocytes) or CD4^−/−^ mice (deficient in CD4^+^ T lymphocytes), thus attributing a neuroprotective role to the CD4^+^ T cells, but not to the CD8^+^ T or B cells (102). Accordingly, the reconstruction of the CD4^+^ T-cell populations following wild-type (WT) bone marrow transplantation to mSOD1/RAG2^−/−^ recipient mice in a stable phase of the disease conferred neuroprotection and increased survival (102).

Interestingly, in a slow initial stage of ALS, neuroprotective activity has been suggested by increasing the levels of CD4^+^CD25^hi^FoxP3^+^ Treg cells. In contrast, the number of circulating Tregs decrease significantly with the rapid progression of the disease. Therefore, it has been suggested that a decrease in Tregs, together with a decrease in FoxP3 expression levels, are associated with short-term survival in ALS patients (103). It should be noted that dysfunction in Treg activity can directly influence the activation of pro-inflammatory microglia, contributing to motor neuron excitotoxicity (104). ALS patients demonstrate an unbalanced ratio among T cell subgroups with an increased number of pro-inflammatory T cells, including Th1 and Th17, and reduced Treg/Th2 lymphocytes (105). In the same way, adoptive transfer of endogenous mSOD1 Tregs during the stable phase of ALS to mSOD1/RAG2^−/−^ recipient mice promoted neuroprotection, postponed the disease onset and considerably increased survival (106).

Recent studies have further highlighted the immunologic alterations in ALS patients and its correlation with disease progression. Proteomic analysis of plasma and peripheral blood mononuclear cells (PBMCs) from ALS patients demonstrated the activation of molecular pathways involved in immunoregulation and cell senescence in faster progressing ALS patients (107). Indeed, Yildiz and colleagues showed that lymphocytes from ALS patients are skewed towards a senescent and late memory state when compared with those from healthy controls (108). Immunosenescence is a process reflecting immune dysfunction, by remodeling of the immune system, with reduction in the number of peripheral blood naïve cells and a relative increase in the frequency of memory cells. This leads to a decline in efficacy of the immune response during aging and is closely correlated with the development of infections, autoimmune diseases, and malignant tumors (109).

In addition, a study comparing 89 newly diagnosed ALS patients in Sweden observed that high frequency of CD4^+^ effector T cells in the blood and cerebrospinal fluid was associated with poor survival, whereas a high frequency of activated Treg in the blood was associated with a better survival (110).

Overall, these studies attributed the neuroprotective capacity of the CD4^+^ T cells to CD4^+^CD25^+^ and CD4^+^CD25^+^FoxP3^+^ Treg cells and suggest that the T cell phenotypes at the time of diagnosis can be good predictors of disease prognosis, as will be described below.

## 4 Immune crosstalk in DMD and ALS: role of regulatory T cells

Migration and maintenance of Tregs in the muscle depends on at least two different mechanisms (1): The first is controlled by the ATP/P2X axis and was initially demonstrated in the mdx mouse model of DMD (111), in which blocking the ATP/P2X7 interaction with periodate-oxidized ATP resulted in an increased number of Treg within the injured muscle. In the absence of ATP/P2X7 signaling, not only do Tregs migrate more intensely to the lesion but muscle damage is reduced, indicating that ATP, released by dying cells, downregulates Treg migration, and that, in the absence of Tregs, muscle damage is more severe (111) (2). Secondly, there is an effect of the IL-33 mediated mechanism of Treg arrival and maintenance within the muscle. IL-33 is an alarmin produced by fibroadipogenic progenitors (FAPs) and skeletal muscle stem cells which can bind to the IL-1 receptor-like 1 protein (also named ST2) present on CD4^+^ T cells (including Tregs), macrophages and FAPs. In fact, FAP-derived IL-33 is crucial for Treg accumulation within the muscle, and consequently, muscle regeneration (**Figure 1A**) (112).

It is well known that both T cells (CD4^+^ or CD8^+^) need to engage their antigen recognition receptor through MHC interaction, so that they can interact specifically with other cells. Studies in mouse models revealed that after transferring muscle specific T cells to a normal recipient, muscle Tregs were only observed within the muscle. In addition, studies in mdx mice have shown that antigen specific Treg cells induced an accelerated rate of regeneration when compared to polyclonal Treg cells (113). Similarly, muscle injury and inflammation were mitigated by the expansion of the Treg cell population, being exacerbated in conditions of Treg cell depletion (114).

As mentioned previously, in ALS, the neuroprotective capacity of the CD4^+^ T cells has been attributed to the CD4^+^CD25^+^FoxP3^+^ Treg cell fraction. It was observed that Tregs in the slow phase of the disease can support a microglial anti-inflammatory phenotype and the expression of neurotrophic factors in the spinal cord through IL-4 secretion. In parallel, they suppress effector T cell proliferation via the secretion of IL-4, IL-10 and TGF-β (115). Likewise, when mSOD1 Treg isolated from spleen and lymph nodes are co-cultured with mSOD1 adult microglia they inhibit NOX2 production and induce NO synthase expression, through IL-4 secretion (115). Thus, infiltration of CD4^+^CD25^+^FoxP3^+^ Tregs in the CNS suppresses neuroinflammation and promotes activation of protective microglia in murine models of ALS (116), with an up-regulation of anti-inflammatory cytokines with a predominance of a neuroprotective phenotype (106).

In contrast, Tregs isolated from mSOD1 mice at a later stage of the disease fail to prolong survival of mSOD1/RAG2^−/−^ recipient mice (106). As the disease progresses, the level of pro-inflammatory cytokines increases, while the number of Treg declines, possibly due to the loss of FoxP3 expression in Treg cells (106). For instance, increased TNF-α release by Th1 and Th17 cells induces Treg dysfunction by inhibiting FoxP3 phosphorylation (96). Moreover, elevated levels of IL-6 released by Th1 cells inhibit the development of FoxP3^+^ Tregs (117), leading to the loss of the ability of Treg cells to sustain an anti-inflammatory response in the microglia, exacerbating a harmful phenotype (**Figure 2**).

Similar to what has been reported in mice, in ALS patients the endogenous Treg cells increase at an early slowly-progressing disease stage. During the course of the disease the development of Tregs is inhibited and their number decreases in parallel to a rapid ALS progression, likely due to the loss of FoxP3 (106, 118). Moreover, in patients, specific Treg subtypes have been correlated with the rate of ALS progression. In the blood of ALS patients with rapid disease progression, there is a reduction in the number of CD45RO^+^ Tregs, the memory cells, which are classified as functionally active with higher levels of FoxP3 expression, when compared to the resting naïve CD45RA^+^ Treg cells (116). Moreover, the rate and severity of ALS progression was inversely correlated with the total Treg counts, total FoxP3^+^ Treg counts and effector CD45RO^+^ Treg counts, while the number of resting CD45RA^+^ Treg did not correlate with disease progression (116).

In addition to this reduction in number, Tregs from patients with rapid disease progression have an impaired ability to inhibit the proliferation of their corresponding effector T cells, when compared to those of patients with slow disease progression, or with healthy controls (103). The Treg suppression capacity was correlated with FoxP3 mRNA and protein levels, indicating that a stable expression is essential to maintain their regulatory capacity (103).

One major problem in the treatment of ALS is that to be successful, the treatment needs to be administered at the time of diagnosis. At present it is estimated that there is a delay of more than one year between disease onset and clinical diagnosis (119), and most of the patients are currently diagnosed at mid stage of progression where reversing neurodegeneration may be very difficult, demonstrating the importance of a predictive factor for the disease. For this reason, increasing the number and function of Treg cells seems like a good therapeutic strategy and some clinical trials aim to target the Treg cells, either by promoting their expansion *in vivo* or by *in vitro* functional stimulation followed by autologous transplantation, as a way of improving quality of life of ALS patients (104). One trial ongoing is MIROCALS, which is a phase II study of Low-dose IL-2 (Ld-IL-2) as a Treg Enhancer for Controlling Neuroinflammation in Newly Diagnosed ALS Patients (https://clinicaltrials.gov/study/NCT03039673?a=1).

## 5 Thymus defects underlying peripheral T cell biology abnormalities in DMD and ALS

The thymus is a primary lymphoid organ responsible for the maturation and differentiation of T lymphocytes (120, 121). During this process, key maturation events correspond to the positive and negative selection, both driven by interactions between developing thymocytes and microenvironmental cells (particularly thymic epithelial cells (TECs), involving both the MHC complex and TCR, respectively present in microenvironmental components and developing thymocytes (122). Negative selection largely prevents autoimmunity and is directly under the control of the transcription factors Aire and Fezf2 expressed by TEC in the medullary region of the thymic lobules (121). Negative selection induces thymocyte death by apoptosis; positive selection rescues developing thymocytes from death and induces their progression towards CD4^+^ or CD8^+^ SP thymocytes, which eventually leave the organ to colonize the peripheral lymphoid organs (123). Lastly, the intrathymic production of central Tregs and subsequent export towards the periphery of the immune system further prevents the development of autoimmunity. Accordingly, central Treg deficiency is related to autoimmune events (124).

Interestingly, the thymus may play a role in the skeletal muscle regeneration process. A study carried out in a model of induced skeletal muscle injury showed that the mean cross-sectional area of regenerating myofibers and the number of myofibers in 8–10 weeks of age thymectomized mice were impaired compared to euthymic controls (125). In addition to this defect in muscle regeneration, thymectomized mice also showed decreased levels of myoblast determination proteins, MyoD 1 and myogenin (MyoG), important myogenic factors for skeletal muscle regeneration. This study further showed that the number of Pax7-positive satellite cells in the muscle was increased in the injured control, compared to thymectomized mice, providing evidence that the thymus can regulate the number of satellite cells during this process of muscle degeneration and regeneration. Interestingly, the authors showed *in vitro* that thymocyte-conditioned medium promoted the proliferation of satellite cells. The CD3^+^CD4^+^CD8^+^ thymocyte subset significantly induced proliferation in satellite cells, although CD4^+^CD8^−^ and CD4^−^CD8^+^ SP thymocytes also displayed a significant effect compared to the control. In contrast, Treg cells had no significant effect in a muscle injury model of thymectomized mice (125). In view of the above data, it seems that the thymus plays a role in the skeletal muscle by its influence on regeneration.

Pioneer studies had previously revealed a thymic atrophy in mdx dystrophic mice, with a decrease in thymocyte numbers, densification of the TEC network and increase in ECM contents. Of note, the peak of thymocyte depletion coincided with the stage of disease severity (126). These findings have recently been confirmed and expanded by Farini and coworkers, who showed that the disorganized thymic morphology was correlated with an abnormal intrathymic T cell development and changes in the maturation stages of DN thymocytes. These events coincide with an increase in the relative numbers of SP cells and Tregs in the thymus of mdx mice compared to controls (127). In addition, the authors showed that transplanting the involuted thymus of mdx animals into nude mice, resulted in a significant increase in the expression of the transcription factors RORγt and T-bet, as well as an increase in the numbers of CD4^+^ and CD8^+^ T cells infiltrating the muscle, compared to nude animals that received the thymus of healthy donors. These results indicate that the process of thymic involution may be a determining factor in DMD pathophysiology and one of the postulated mechanisms was a dysregulation in the release of autoimmune or Treg cells (33).

Despite the lack of studies that aim to characterize in detail the role of the thymus in ALS, evidence suggests that thymic function is compromised in ALS-SOD1 patients and in the mSOD1 mouse model. In 2010, Seksenyan and colleagues demonstrated a thymic defect in parallel to the MN dysfunction in mSOD1 mice and ALS patients. In this study, the authors observed a reduction in the absolute number of total thymocytes in mSOD1 mice. This reduction was reflected in all thymocyte populations, defined by the expression of CD4 and CD8 coreceptors, i.e., CD4^-^CD8^-^ DN, CD4^+^CD8^+^ DP, CD4^+^CD8^-^ and CD4^-^CD8^+^ SP cells. These changes were also observed in ALS patients at the final disease stage. Moreover, sections of the thymus of mSOD1 mice in advanced stages of the disease stained with hematoxylin and eosin, showed a loss of definition in the cortical and medullary regions (128), which may reveal an abnormal thymic architecture during ALS progression and, consequently, impaired T cell development. Indeed, the analysis of signal joint T-cell receptor rearrangement excision circle (sjTREC) in PBMCs from ALS patients, revealed decreased numbers of sjTREC compared to healthy controls regardless of the age of the patients, possibly indicating a reduction in the export of naïve T cells from the thymus of ALS patients (128).

## 6 Concluding remarks

The data discussed above provide evidence concerning the complexity of the cellular and molecular interactions involving the immune system in two very different neuromuscular disorders, DMD and ALS, which may reflect similar defects common to other neuromuscular diseases. Distinct types of interactions have been described, comprising among others cytokine/chemokine production, cell-cell and cell-matrix interactions between T lymphocytes and other cells of the immune system with target cells of the muscular or nervous tissues. Although this reveals the complexity of the system, it also can be envisioned as a window of opportunity to design novel therapeutic strategies (including synthetic moieties, cell and gene therapy, as well as immunotherapy) by acting upon one or more targets. In this respect, ongoing clinical trials on DMD patients through VLA-4 inhibition, and in ALS, focusing on regulatory T cells, have both shown promising results.

## Author contributions

JL and WS made extensive review of the literature listed and elaborated different sections of the review. JL, LT, WS, DM-d-C, and PS drafted and drew the figures. JL, WS, and PS conceived, designed and supervised the review. VM, GB-B, and DM-d-C edited the manuscript. All authors contributed to the article and approved the submitted version.
